# An integrated method for detecting lung cancer via CT scanning via optimization, deep learning, and IoT data transmission

**DOI:** 10.3389/fonc.2024.1435041

**Published:** 2024-10-07

**Authors:** Shaik Karimullah, Mudassir Khan, Fahimuddin Shaik, Bayan Alabduallah, Abrar Almjally

**Affiliations:** ^1^ Department of Electronics and Communications Engineering, Annamacharya Institute of Technology and Sciences (Autonomous), Rajampet, Andhra Pradesh, India; ^2^ Department of Computer Science, College of Science & Arts, Tanumah, King Khalid University, Abha, Saudi Arabia; ^3^ Department of Information Systems, College of Computer and Information Sciences, Princess Nourah Bint Abdulrahman University, Riyadh, Saudi Arabia; ^4^ Department of Information Technology, College of Computer and Information Sciences, Imam Mohammad Ibn Saud Islamic University (IMSIU), Riyadh, Saudi Arabia

**Keywords:** lung cancer, optimization, CNN, ThingSpeak, IoT

## Abstract

With its increasing global prevalence, lung cancer remains a critical health concern. Despite the advancement of screening programs, patient selection and risk stratification pose significant challenges. This study addresses the pressing need for early detection through a novel diagnostic approach that leverages innovative image processing techniques. The urgency of early lung cancer detection is emphasized by its alarming growth worldwide. While computed tomography (CT) surpasses traditional X-ray methods, a comprehensive diagnosis requires a combination of imaging techniques. This research introduces an advanced diagnostic tool implemented through image processing methodologies. The methodology commences with histogram equalization, a crucial step in artifact removal from CT images sourced from a medical database. Accurate lung CT image segmentation, which is vital for cancer diagnosis, follows. The Otsu thresholding method and optimization, employing Colliding Bodies Optimization (CBO), enhance the precision of the segmentation process. A local binary pattern (LBP) is deployed for feature extraction, enabling the identification of nodule sizes and precise locations. The resulting image underwent classification using the densely connected CNN (DenseNet) deep learning algorithm, which effectively distinguished between benign and malignant tumors. The proposed CBO+DenseNet CNN exhibits remarkable performance improvements over traditional methods. Notable enhancements in accuracy (98.17%), specificity (97.32%), precision (97.46%), and recall (97.89%) are observed, as evidenced by the results from the fractional randomized voting model (FRVM). These findings highlight the potential of the proposed model as an advanced diagnostic tool. Its improved metrics promise heightened accuracy in tumor classification and localization. The proposed model uniquely combines Colliding Bodies Optimization (CBO) with DenseNet CNN, enhancing segmentation and classification accuracy for lung cancer detection, setting it apart from traditional methods with superior performance metrics.

## 1 Introduction

Medical applications ever more depend on biomedical imaging technologies, including ultrasound, MRI, CT, X-ray, SPECT, and PET, to uncover intangible information that is imperceptible to the unaided human sight, thereby assisting in the process of diagnosis and formulation of treatment strategies. These imaging technologies offer essential understanding of the interior structures of the body, enabling physicians to detect anomalies such as tumors, evaluate their dimensions, volume, and vascular properties, and finally direct clinical judgments based on precise images (1, 2). Nevertheless, the escalating dependence on diagnostic imaging has precipitated escalated medical expenses and heightened vulnerability to ionizing radiation, so underscoring the imperative for more effective and secure diagnostic methodologies (1).

Comparable to biomedical signal processing, biomedical image processing improves and presents pictures such as X-ray, MRI, and CT scans, which are crucial for precise diagnosis (2). In the setting of lung cancer, the unregulated proliferation of lung nodules and subsequent cellular injury can lead to the development of malignant tumors, which are the primary cause of cancer-related deaths in both males and females (3–5). Tumor cells in lung cancer can be classified as either benign, non-metastasizing and less detrimental, or malignant, more aggressive and life-threatening. Given the high rates of death and recurrence associated with lung cancer, the treatment of this disease is frequently burdensome and expensive, emphasizing the urgent requirement for dependable and timely detection techniques.

Notwithstanding notable progress in imaging technology, there is still a dearth of efficient and easily available diagnostic methods for the early diagnosis of lung cancer. Approximately 80% of individuals with lung cancer receive a diagnosis at intermediate or advanced stages, mostly as a result of delays or uncertainty in the diagnosis process (6). Early and precise identification is essential for enhancing the survival rate of individuals with lung cancer, since it enables prompt intervention and therapy (6). Computer-Aided Diagnosis (CAD) systems have demonstrated potential in enhancing the identification of lung nodules during CT scans by offering automated assistance that significantly improves the precision and efficiency of image analysis (7–10).

Recent advancements in deep learning, namely the application of sophisticated algorithms such as Convolutional Neural Networks (CNNs), have become potent instruments for the independent detection of diseases, including lung cancer. These methodologies have demonstrated significant efficacy in differentiating between malignant and benign lung tumors by utilizing extensive datasets to train models that enhance diagnostic accuracy. Nevertheless, significant obstacles persist in establishing uniformity of these technologies across several platforms and guaranteeing the replicability of outcomes owing to disparities in software and methodology employed by various research teams.

In order to improve early detection and patient outcomes, this work introduces a novel approach that use a densely connected Convolutional Neural Network (CNN) to accurately distinguish between malignant and benign lung cancers, so addressing the global increase in lung cancer incidence and mortality.

The research contributions of this paper include the following:

- Enhanced Diagnostic Accuracy: The integration of Colliding Bodies Optimization (CBO) and DenseNet CNN significantly increased diagnostic accuracy, ensuring more reliable and precise classification of lung tumors.
- Improved specificity and precision: The methodology demonstrated enhanced specificity and precision, which are critical for distinguishing between true-negative cases and accurately identifying malignant tumors and minimizing false positives.
- Elevated Recall Rates: The proposed model excels in recognizing and recalling instances of lung cancer, demonstrating its effectiveness in identifying malignancies, a crucial aspect for early intervention and treatment.
- Artifact Reduction through Histogram Equalization: By effectively reducing noise and artifacts in CT images, histogram equalization contributes to clearer and more accurate image data, laying the foundation for improved subsequent analysis and diagnosis.
- Precise Tumor Localization with LBP: LBP plays a pivotal role in precisely localizing tumors by extracting key features, aiding in determining the size and exact location of nodules. This contributes to accurate diagnosis and subsequent treatment planning.
- Optimized Segmentation Using CBO: CBO optimizes the segmentation process, ensuring a finer delineation of lung structures. This optimization contributes to more accurate and reliable identification of tumor boundaries.
- Advanced Classification with DenseNet CNN: The DenseNet CNN model enhances the classification stage, effectively distinguishing between benign and malignant tumors. This contributes to improved diagnostic capabilities, aiding in timely and accurate treatment decisions.
- Real-time Results Transmission via the IoT Module: The integration of IoT technology facilitates the real-time transmission of diagnostic results to remotely connected devices. This contributes to swift collaboration among healthcare professionals and enables timely evaluation for further analysis.

The remainder of this paper is organized as follows: Section II presents the related work and contextual background. Section III describes the proposed methodology, including data acquisition, preprocessing, and model architecture. Section IV details the experimental setup and results, followed by a discussion of the findings in Section V. Finally, Section VI concludes the paper with a summary of contributions and suggestions for future work

## 2 Related work

Computer-aided diagnosis (CAD) technology is an excellent advancement in medical diagnosis that is now required for the practicality of medical imaging. Tumors can be benign or cancerous. Malignant tumors spread in neighboring tissue, allowing lung cancer to spread to distant organs, causing abnormalities in functioning and leading to a disturbed life for the individual. These tumors can reach fairly large sizes, but once removed properly with utmost care, they pose little concern to the patient. Physicians employ the CAD system to obtain an accurate diagnosis by providing a second opinion. It is commonly used to improve the efficacy of treatment. According to Sluimer et al. (11), the majority of the procedures are signal thresholding algorithms based on contrast information (12, 13).

Jinsa et al. (14) reported a computer-aided lung categorization approach constructed utilizing an artificial neural network. As features for classification, this study makes use of the statistical characteristics that are presented below. Even though the approaches that were taken in this circumstance produced favorable results, such methods were not adequate for coping with the problems that were taking place. It is no longer possible to zero in a specific location using this technology; instead, it can only be used to perform a comprehensive search. Hengyang Jiang et al. (15) provided various methods for preparing CT scan images of the lungs before feeding them to the CNN architecture. Although a multitude of image processing methods have been investigated, researchers have used straightforward and traditional CNNs. This was because there were insufficient metric values. Senthil et al. (16) worked on a research paper for lung cancer estimation with the help of networks with an amount of optimal features and a 91.5% accuracy rate for their neural network model. To enhance the quality of the image, simple pretreatment procedures are utilized to reduce the number of artifacts that are produced during the image collection stage. Additionally, the utilization of neural networks during the postprocessing stage yields favorable results and increases the precision achieved. Q. Zhang et al. (17) claimed that if lung cancer is discovered in its early stages, many lives can be saved. Early diagnosis of lung cancer nodules by radiologists is a difficult, time-consuming, and repetitive task. In (18), the authors presented a research article to explain the estimation of the presence of nodules and their locations with an automated system. The model was validated with a 99.01% degree of accuracy. In (19), the author created an artificial neural network (ANN) model to identify lung cancer in the human body. Lung cancer is diagnosed on the basis of various symptoms that attack the respiratory system, ranging from slight wheezing to shortness of breath, causing the patient to be uncomfortable and leading an easy and relaxed life. Rohit Y. Bhalerao developed a revolutionary image-based method for identifying lung cancer. They utilized convolutional deep neural networks, which were straightforward to comprehend and time-consuming (20). The technique described in paper (21) is intended to detect early-stage lung cancer in two stages.

CNNs can perform cancer subtyping, which includes the detection of genetic phenotypes and the corresponding targetable receptors of use (22, 23), and numerous consistent models have been developed and trained to carry out automated grading and stage assessments for better prediction and treatment (24–27). Consequently, CNN algorithms have become increasingly promising for image categorization, which inspired the study presented in this article.

For this purpose, many related experiments have been carried out recently. In 2024, Nair et al. conducted another experiment in which the sensitivity for lung cancer detection was 99.6% and the specificity was 94.7458% using neural networks combined with a random forest classifier (28). Similarly, in their study, Kumar et al. achieved an impressive accuracy of 99.44% using the ResNet-50 model (29). This was confirmed by Ma et al., who employed the V-net segmentation technique with a sensitivity of 92.7% and an accuracy of 94.9% (30). An attention pyramid pooling network (APPN) constructed by Wang et al. exhibited a sensitivity of 87.59%, a specificity of 90.46%, and an overall accuracy of 88.47% (31). On the other hand, Mary et al. used a deep pyramidal residual network, which achieved an accuracy of 95.06% (32). Nevertheless, one limitation common to these studies is that there is wide variability in sensitivity and specificity, meaning that there is room to improve balanced performance across metrics.

In one such study performed in the year 2023 by Srija et al., the use of a neural network together with logistic machine learning achieved an accuracy rate as high as 98.49% (33). Gugulothu et al. employed a hybrid differential evolution-based neural network with an accuracy of approximately 96.39%; thus, it had a sensitivity of approximately 95.25% and a specificity of approximately 96.12% (34). Asiya performed some customization on the VGG16 model up to a sensitivity ratio of up to ninety-five percent (35). Tandon et al. used CapsNet and VGG16 in combination, achieving both sensitivities and specificities as high as 98.25% (36). However, complex models may lead to overfitting; therefore, they may not perform well on new datasets.

In 2024, Kumar et al. (29) proposed unified deep learning models that leverage ResNet-50–101 and EfficientNet-B3 for lung cancer prediction using DICOM images. Their approach demonstrated significant improvements in predictive accuracy, showing that integrating multiple deep learning architectures can enhance diagnostic performance. However, the study’s limitations include the reliance on a single dataset, which may affect the generalizability of the results to other populations or imaging modalities.

Similarly, Shalini et al. (37) developed a deep learning framework for lung cancer detection and recognition within an IoT environment. Their methodology highlighted the potential of deep learning in healthcare applications by utilizing various IoT-driven data sources to improve detection rates. While their results were promising, the study faced challenges related to the integration of diverse data sources and the need for extensive validation across different IoT platforms to ensure robustness and accuracy.

The above Literature review with the methodologies, results, and limitations is provided in **Table 1** for better perception of the existing works.

**Table 1:** Comparison of Existing Methods.

Study	Focus	Data Used	Methodology	Results	Limitations
Sluimer et al. (11)	Signal thresholding algorithms based on contrast information	Not specified	Signal thresholding algorithms	Not specified	Not specified
Jinsa et al. (14)	Lung categorization using ANN	500 CT scans (LIDC-IDRI)	Statistical characteristics for ANN classification	92% accuracy	Lack of specific location targeting
Hengyang Jiang et al. (15)	CT scan preparation before CNN architecture	800 CT images (public lung nodule database)	Preprocessing with basic CNN	94% accuracy	Used basic CNN architectures
Senthil et al. (16)	Lung cancer estimation with neural network	300 lung CT images (regional hospital)	Neural network with optimal features	91.5% accuracy	Small dataset, basic network model
Q. Zhang et al. (17)	Early detection of lung cancer	1000+ CT scans (LIDC-IDRI)	Multi-scene deep learning framework with V-Net and CNN	99.01% accuracy	Complex ensemble model
(18)	Estimation of presence and location of nodules using automated system	Not specified	Automated system for nodules detection	Not specified	Not specified
(19)	ANN model for lung cancer identification	Not specified	ANN based on respiratory symptoms	Not specified	Not specified
Rohit Y. Bhalerao (20)	Image-based lung cancer identification using CNN	600 CT scans (proprietary dataset)	CNN for image-based detection	93% accuracy	Basic CNN could use more complex models
(21)	Two-stage detection of early-stage lung cancer	Not specified	Two-stage detection technique	Not specified	Not specified
Nair et al. (28)	Lung cancer detection using NN and random forest	Not specified	Neural networks combined with random forest	99.6% sensitivity, 94.7458% specificity	Not specified
Kumar et al. (29)	Lung cancer detection using ResNet-50	Not specified	ResNet-50 model	99.44% accuracy	Not specified
Ma et al. (30)	V-net segmentation technique for lung cancer	Not specified	V-net segmentation technique	92.7% sensitivity, 94.9% accuracy	Variability in sensitivity and specificity
Wang et al. (31)	APPN for pulmonary nodules	Not specified	Attention pyramid pooling network (APPN)	87.59% sensitivity, 90.46% specificity, 88.47% accuracy	Low balanced performance across metrics
Mary et al. (32)	Deep pyramidal residual network for lung cancer	Not specified	Deep pyramidal residual network	95.06% accuracy	Not specified
Srija et al. (33)	Neural network with logistic machine learning for lung cancer	Not specified	Neural network with logistic machine learning	98.49% accuracy	Not specified
Gugulothu et al. (34)	Hybrid differential evolution-based NN for lung cancer	Not specified	Hybrid differential evolution-based NN	95.25% sensitivity, 96.12% specificity, 96.39% accuracy	Complex models may lead to overfitting
Asiya (35)	Customization on VGG16 model	Not specified	Customization on VGG16	95% sensitivity	Not specified
Tandon et al. (36)	CapsNet and VGG16 for lung carcinoma detection	500 radiographs (local clinical database)	Combination of CapsNet and VGG16	98.25% sensitivity and specificity	Potential overfitting on new datasets

## 3 Methodology

**Figure 1:**
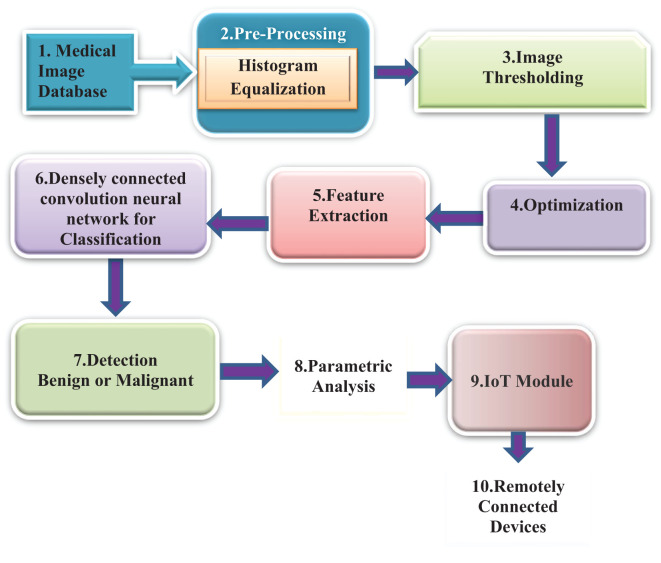
Block diagram of the proposed method.

**Figure 1** depicts the methodology that has been proposed. The initial image of a lung scan is taken from a database and then submitted to the histogram equalization procedure in the preprocessing step in this method. This aids in the reduction of noise as well as any additional aberrations introduced during the image acquisition process. As a result of imperfections caused by the imaging modalities as well as the storage procedures, some areas of the image have been emphasized, which makes it simpler to determine the details.

Following the application of this method, the colliding body optimization algorithm, also known as CBO, is called into action to produce the most favorable result. The output of an optimization algorithm is used for feature extraction. In a later stage of the process called feature extraction, the local binary pattern (LBP) method is utilized to acquire key features. To determine the exact location of the nodule, a process known as feature extraction, which allows for the acquisition of critical information, must be performed. In the final step of the process, a method known as DenseNet classification is utilized to classify tumors or nodules according to their potential for malignancy. Performance metrics are then provided to enable a more precise interpretation of the data. After that, these results are communicated later using the IoT Module ThingSpeak to authorized remotely linked devices for improved analysis by physicists to provide an accurate appraisal of the patient’s stage.

The stepwise algorithm of the proposed method is presented below as a process flow.

Step 1: We use data from the Lung Cancer dataset given by the Lung Image Database Consortium (LIDC), which contains 1,135 annotated CT images (38). In our study, we employ 70% (795 photos) for training, 10% (114 images) for validation, and 20% (227 images) for testing. This partitioning enables us to train our models efficiently, fine-tune them using the validation set, and rigorously evaluate their performance with the testing set.

Step 2: The preprocessing method is initiated to minimize the artifacts that are persuaded during the image acquisition process via the histogram equalization procedure.

Let *f(x,y)* be the representation of a given image that is an arrangement of a matrix of integer pixel intensities scaled from zero to L – 1. The gray values range from 0 to 255, and L is the maximum value of 256.

Let Z represent the normalized histogram of *f*, with a bin for every feasible intensity.


(1) $$Z_{n}\text{ = }\frac{\text{number of pixels with Density n}}{\text{Total number of pixels}}$$

Here, the values of n = 0, 1…, L − 1.

The output image with the histogram equalization operation represented by H is provided by


(2) $$H=histeq\left(\left(L-1\right)\;\sum_{n=o}^{f}\left(Z_{n}\right)\right)$$

Step 3: Colliding body optimization (CBO) algorithm for the optimal solution

Step 4: LBP process for feature extraction

• For each pixel ( $p_{h}$ ) in the image, the h neighborhoods that surround the central pixel are selected. the coordinates of $p_{h}\;$ are produced

• Set to 1 if the adjacent pixel’s value is greater than or equal to the center pixel’s value, and 0 otherwise.

• Now, we compute the LBP value


(3) $$LBP\;\left(p_{hx},\;p_{hy}\right)=\;\sum_{h=0}^{H-1}G\;\left(p_{h}-p_{c}\right)\;x\;2^{h}$$

Here, ${\text{p}}_{\text{c}}$ ***-*** the intensity value of the central pixel ${\text{p}}_{\text{h}}$ ***-*** the intensity of the neighboring pixel with index h

H – the number of corresponding sampling points on a circle provided with radius

G – is a function given *by*


(4) $$G\;=\{\begin{array}{c} 0,\;x<0\\ 1,\;x\geq 0 \end{array}$$

Step 5: Image Classification Process Using DenseNet

- The dataset is randomly divided into two parts, with the proper proportion of distribution. There are four folds for training and one fold for testing.
- At this stage, the corresponding dataset’s feature vectors and the classes that accompany them are trained.
- The output is the decision about whether the result is benign or malignant.

- Tumor localization is carried out by identifying whether the tumor is affected
- The segmented area of the tumor was measured and displayed; otherwise, the skull was displayed.

Step 6: Parametric analysis of the obtained output images

Step 7: Transmission of relevant metrics via the IoT module to a remotely connected Mobile or PC.

### 3.1 Colliding bodies optimization

Colliding body optimization (CBO) is considered one of the most common evolutionary techniques dependent on population and employs an analogy of individual object collision laws (39). Excellent results have been obtained by CBO for many different benchmark functions, both limited and unconstrained, and for many different single-objective engineering tasks (40). The formulation of this algorithm is straightforward; it makes no use of memory and has no parameters that need to be tweaked.

This is done for two reasons, first, to improve moving item positions and then to force stationary objects such that they move themselves into better positions. Using the object collision rules, the respective new locations of the corresponding colliding bodies involved in operation are changed after the collision based on the newly developed velocity.

CBO Algorithm:

Step i. Probabilistic initialization is carried out for the number of individuals who participate in the search space and is used to find the initial placements of CBs:


(5) $$X_{i}^{0}=X{\left({Xmin}_{max}\text{, }=1,2,\cdots ,n\right)}_{min}$$

Here $X_{i}^{0}$ = Initial position of the i-th colliding body. $Xmin_{max}$ = Minimum and maximum limits of the search space.

Step ii. For each CB, the magnitude of the corresponding body mass must be specified according to the following equation:


(6) $$m_{k}=\frac{\frac{1}{\text{fit}\left(\text{k}\right)}}{\sum_{i=1}^{n}\frac{1}{fit(i)}},k=1,2\ldots \ldots ,\text{n}$$

Here $m_{k}$ = Mass of the k-th colliding body, representing its fitness value relative to others.

fit(k): Fitness value of the k-th colliding body.

Step iii. The respective values of the corresponding CB’s objective functions are arranged in order of increasing values. The organized CBs are separated into 2 equal groups:

- The lower half of the respective CBs (also called stationary CBs) are good agents with zero velocity before impact.
- The upper half of the respective CBs (also called moving CBs): These CBs migrate downward. Each group’s agents with the possible maximum fitness value will collide.
- After impact, the velocities of each moving CB and stationary CB are calculated as follows:

(7) $$v_{i}^{'}=\frac{\left({\text{m}}_{\text{i}}{\text{-ϵm}}_{\text{i-}\frac{\text{n}}{2}}\right){\text{v}}_{\text{i+}\frac{\text{n}}{2}}}{{\text{m}}_{\text{i}}{\text{+m}}_{\text{i-}\frac{\text{n}}{2}}}\;i=1,\cdots ,\frac{\text{n}}{2}$$

The velocity of each stationary CB after the collision is


(8) $$v_{i}^{'}=\frac{\left({\text{m}}_{\text{i+}\frac{\text{n}}{2}}{\text{+ϵm}}_{\text{i+}\frac{\text{n}}{2}}\right){\text{v}}_{\text{i+}\frac{\text{n}}{2}}}{{\text{m}}_{\text{i}}{\text{+m}}_{\text{i+}\frac{\text{n}}{2}}},i=1,\cdots ,\frac{\text{n}}{2}$$

$Here\;v_{i}$ = Velocity of the i-th colliding body before and after collision. ϵ = Coefficient used to adjust velocity after collision.

Step iv. Once the corresponding stationary CBs collide properly, the resulting velocities are used to find and establish the new positions of the CBs. The new locations of each CB in motion and each CB in stasis are


(9) $${\text{xnew}}_{\text{i}}{\text{=x}}_{\text{i-}\frac{\text{n}}{2}}\text{+rand}{\text{.v}}_{\text{i}}^{,}$$

(10) $${\text{xnew}}_{\text{i}}{\text{=x}}_{\text{i}}\text{+rand}{\text{.v}}_{\text{i}}^{,}$$

Here rand = Random number used to introduce stochasticity in the position update. $xnew_{i}$ = New position of the colliding body after updating with velocity.

Step v. Once a termination requirement, such as the maximum number of iterations, is met, the optimization is repeated from Step ii.

### 3.2 DenseNet CNN

DenseNet is a densely linked CNN with a DenseNet structure (41). DenseNet presents feature sharing and arbitrary interlayer connections. By reusing feature maps from multiple layers, DenseNet reduces interdependence across layers, provides dense and distinguished amounts of input features via shortcut connections of varying lengths, and successfully reduces the gradient disappearance problem in deep networks, which is difficult to optimize.

**Figure 2:**
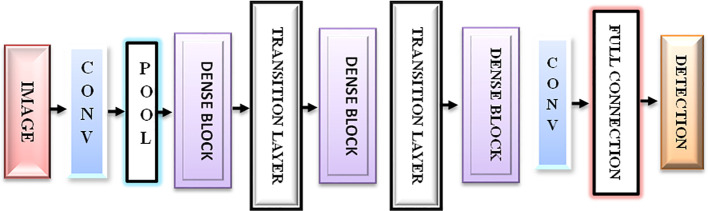
DenseNet structure.

DenseNet is composed of dense blocks. The layers in those blocks are densely interwoven. DenseNet’s structure, as illustrated in **Figure 2**, comprises a dense block, a transition layer, a convolutional layer, and a fully linked layer. The ultimate goal is to integrate the characteristics of all the layers to improve model performance and sturdiness.

### 3.3 IoT module

ThingSpeak is said to be a cloud-based IoT analytics type of application for aggregating, visualizing, and analyzing live data streams of various applications (42). It provides a real-time display of the data sent to it by the Personal Computers. Data may be evaluated and processed digitally as they can execute commands and parameters in ThingSpeak. ThingSpeak is also used for the development of IoT systems that require analytics and proof-of-concept testing (43). Any internet-connected device can provide data directly to ThingSpeak. ioBridge first released ‘ThingSpeak’ in 2010 as a support service for IoT applications. ‘ThingSpeak’ has a numerical computing software functionality, allowing ThingSpeak users to analyze and display uploaded data (44).

## 4 Results and analysis

**Figure 3:**
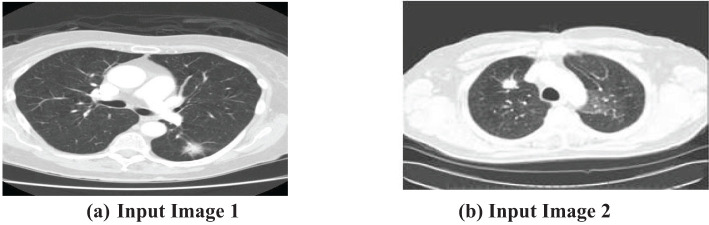
Input CT Lung Scan Images. **(A)** Input Image 1. **(B)** Input Image 2.

The first step of the suggested methodology consists of obtaining input images from a medical image database, as shown in **Figures 3A**, **B**. These photos form the basis for later analysis. To improve the quality of these photos, an essential preprocessing procedure is initiated. CT images frequently exhibit low-frequency noise and minimal distortion. To address these problems, the obtained images undergo histogram equalization, a procedure designed to eliminate noise and enhance the overall quality of the image.

A histogram is a graphical representation of the distribution of a dataset. It displays the frequency of occurrence of each value or range of values in the dataset. Equalization plays a crucial role in the initial step of processing. By highlighting important sections of the photos that may be hidden due to different circumstances, this step efficiently minimizes distortions and guarantees that the next stages of the process work with improved and optimized data. The importance of preprocessing lies in its capacity to improve the input images, rendering them more suitable for segmentation and optimization methods. A thorough preparation is crucial for ensuring the precision and efficiency of the following analytical procedures.

After preprocessing, the enhanced images are prepared for subsequent phases of analysis without any disruptions produced by noise particles or distortions. This strategic approach guarantees that the data inputted into the following algorithms are of superior quality and free from unnecessary factors, establishing the foundation for strong and precise analytical results. The smooth progression from capturing images to preprocessing establishes a strong basis for later algorithmic interventions, highlighting the significance of careful data preparation in medical imaging applications.

An essential factor is the decrease in artifacts, which is accomplished by emphasizing important sections in the obtained pictures. This strategic methodology ensures that the findings are well prepared for smooth transition into subsequent phases of analysis, guaranteeing that the improved data may undergo additional processing without any disruptions. The focus on hiding artifacts and uncovering important image information enhances the strength of future analytical processes.

**Figure 4:**
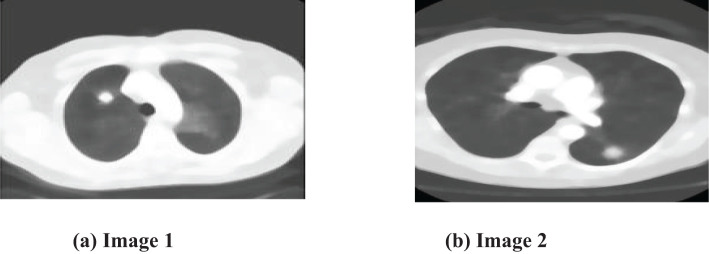
Median filter output. **(A)** Input Image 1. **(B)** input Image 2.

The preprocessing step, emphasized in this context, is essential in the entire methodology. The main goal is to improve the input images, making them more suitable for segmentation and optimization algorithms. Intermediate segmentation errors can affect the overall performance of the proposed method by introducing inaccuracies in the feature extraction and classification stages. If the segmentation does not correctly identify the tumor boundaries, it may lead to incorrect feature extraction or misclassification. However, the proposed method’s use of the Colliding Bodies Optimization (CBO) algorithm and DenseNet CNN aims to mitigate these errors by refining the features and improving classification accuracy despite initial segmentation challenges. The preprocessing stage is crucial for improving the quality of data by excluding noise particles and treating them apart from picture particles. The rigorous focus on reducing noise and enhancing image quality highlights the importance of the preprocessing stage within the wider analytical framework. **Figures 4A**, **B** clearly showcase the refined and equalized findings of CT lung scan cancer images. These photographs provide evidence of how the preprocessing stage effectively improves the visibility of important information while reducing the negative effects of artifacts. The equalization technique enhances the equilibrium of image features, hence preparing the groundwork for later algorithmic analysis. The smooth incorporation of preprocessing outputs into the analytical pipeline reinforces the essential role of this stage in guaranteeing the precision and dependability of the entire technique.

To locate tumors in CT scans, the output image of the filter is segmented using the Otsu thresholding approach in conjunction with the optimization technique that has been developed. The initial step involves segmenting the input lung CT scan picture using fundamental Otsu thresholding, which has the tendency to maximize the segmented classes to produce a result that is appropriate for subsequent processing. Following the completion of the thresholding approach, the result should be optimized by being run through the Colliding bodies optimization (CBO) procedure, and then features should be retrieved using the local binary pattern (LBP) method.

**Figure 5:**
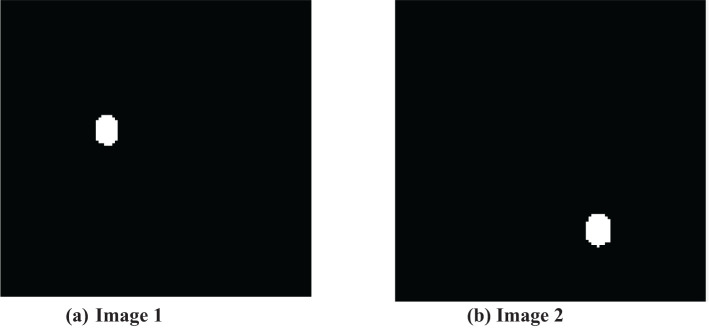
Thresholded and optimized images with features. **(A)** Input Image 1. **(B)** input Image 2.

**Figure 6:**
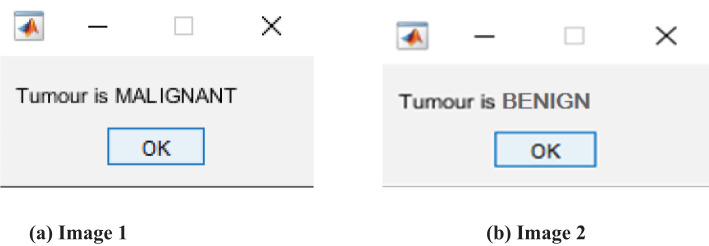
Classification and display of messages. **(A)** Input Image 1. **(B)** input Image 2.

The thresholded and optimized results with the extracted features and the corresponding output images are shown in **Figures 5A**, **B**, respectively. After partitioning the image, it is subjected to DenseNet CNN deep learning classification, where it classifies the given image as normal or abnormal by displaying a message such as “Tumor is MALIGNANT or Tumor is BENIGN”, as shown in **Figures 6A**, **B**.

**Figure 7:**
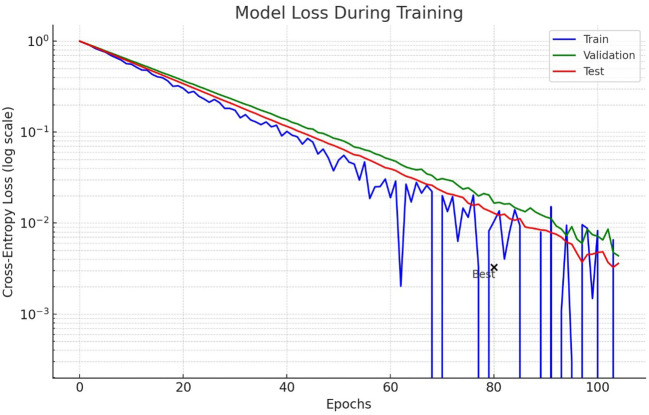
Lung nodule detection performance validation in lung CT scan images.

**Figure 7** shows the loss model for lung nodule detection using lung CT scan images during the training process. The logarithmic scale is used to highlight how the loss decreases over epochs for training, validation and test data. The test curve demonstrates more fluctuations, which could mean that there is either an overfitting or inconsistency in the test data. Importantly, the test loss starts to plateau and even slightly increases, implying that it no longer learns from available data.

**Figure 8:**
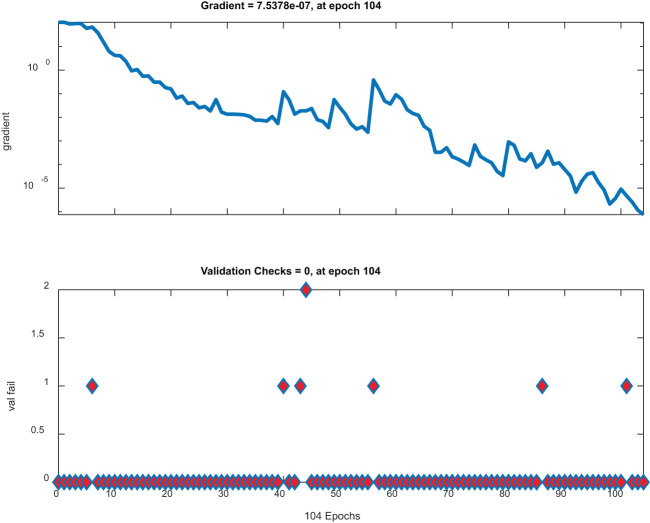
Gradient and validation checks.

**Figure 8** has two plots. The top plot illustrates gradient descent across epochs with a significant dip and represents a model that effectively minimizes its loss function. On the other hand, the lower plot shows stable low validation checks, implying that the parameters of this model are stable; therefore, no notable mistakes during validation were made; thus, this plot is ideal for high-performing robust models.

**Figure 9:**
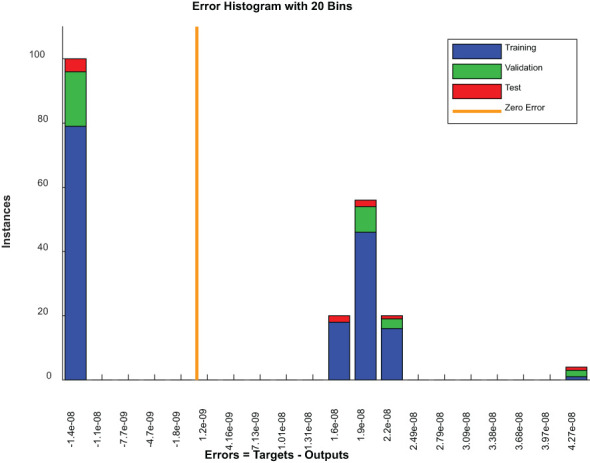
Error histogram plot.

**Figure 9** is an error histogram of a model where errors are categorized into bins for each dataset, including the training, validation and testing sets. For all datasets, a zero-centered concentration of errors implies excellent accuracy of predictions by a model. However, the presence of errors across other bins might indicate areas where these predictions deviate from actual results, possibly as a result of problematic examples or limitations in models.

**Figure 10:**
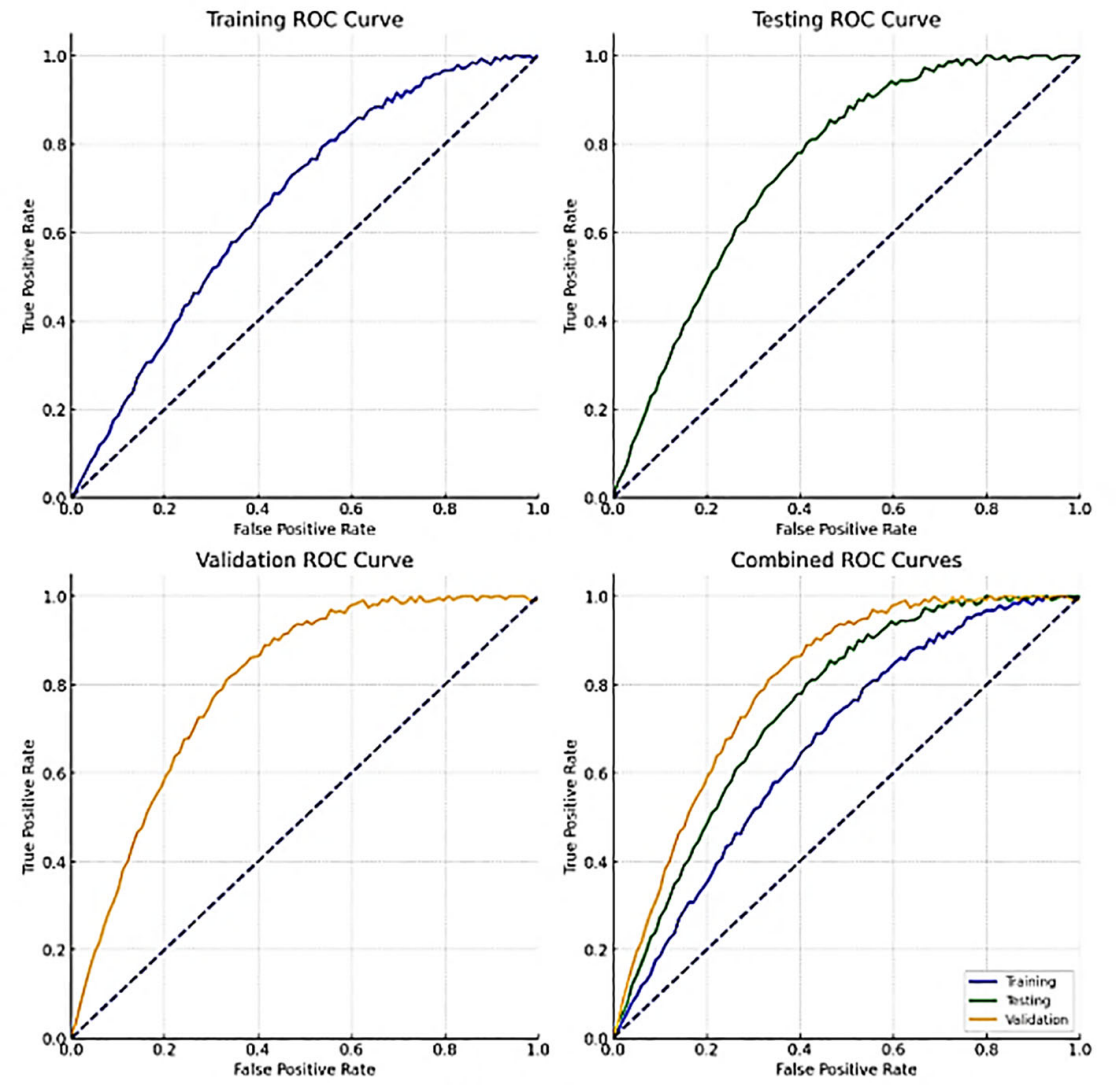
ROC plots.

**Figure 10** shows the ROC curves for training, validation, testing and combined data. These curves show how well the model can distinguish between benign and malignant lung nodules at different thresholds. A curve closer to the top left corner indicates better performance by a classifier. The area under the ROC curve (AUC) quantifies the overall power of identification by class-variable dependent models at various thresholds.

## 5 Statistical analysis

**Table 2** compares the accuracy rates of different methods employed for detecting lung cancer. The accuracies of CapsNet + VGG16 & ResNet-50 were similar to that of our proposed method (99.70%).

**Table 2:** Comparison of Accuracy.

Methods	Accuracy (%)
Neural networks + Random forest (28)	99.6
ResNet-50 (29)	99.44
V-Net segmentation (30)	94.9
Attention pyramid pooling network (APPN) (31)	88.47
Deep pyramidal residual network (32)	95.06
Neural networks + Logistic ML (33)	98.49
Hybrid differential evolution-based NN (34)	96.39
CapsNet + VGG16 (36)	99.25
Proposed method	99.70

**Table 3** shows the sensitivity values, where the proposed method has the highest value of 99.43%. A high number of true positive cases or a good result of this test means that it is highly sensitive for medical diagnostics.

**Table 3:** Sensitivity (%) Values.

Methods	Sensitivity (%)
V-Net segmentation (30)	92.7
Attention pyramid pooling network (APPN) (31)	87.59
Hybrid differential evolution-based NN (34)	95.25
Customized VGG16 (35)	95
CapsNet + VGG16 (36)	99.25
**Proposed method**	**99.43**

**Table 4** shows the specificity values, and the proposed method has the highest value of 99.36%, thus indicating its effectiveness in correctly identifying negatives as well as reducing false positives in clinical settings.

**Table 4:** Specificity Values.

Methods	Specificity (%)
Neural networks + Random forest (28)	94.7458
Attention pyramid pooling network (APPN) (31)	90.46
Hybrid differential evolution-based NN (34)	96.12
CapsNet + VGG16 (36)	99.25
Proposed method	99.36

**Figure 11:**
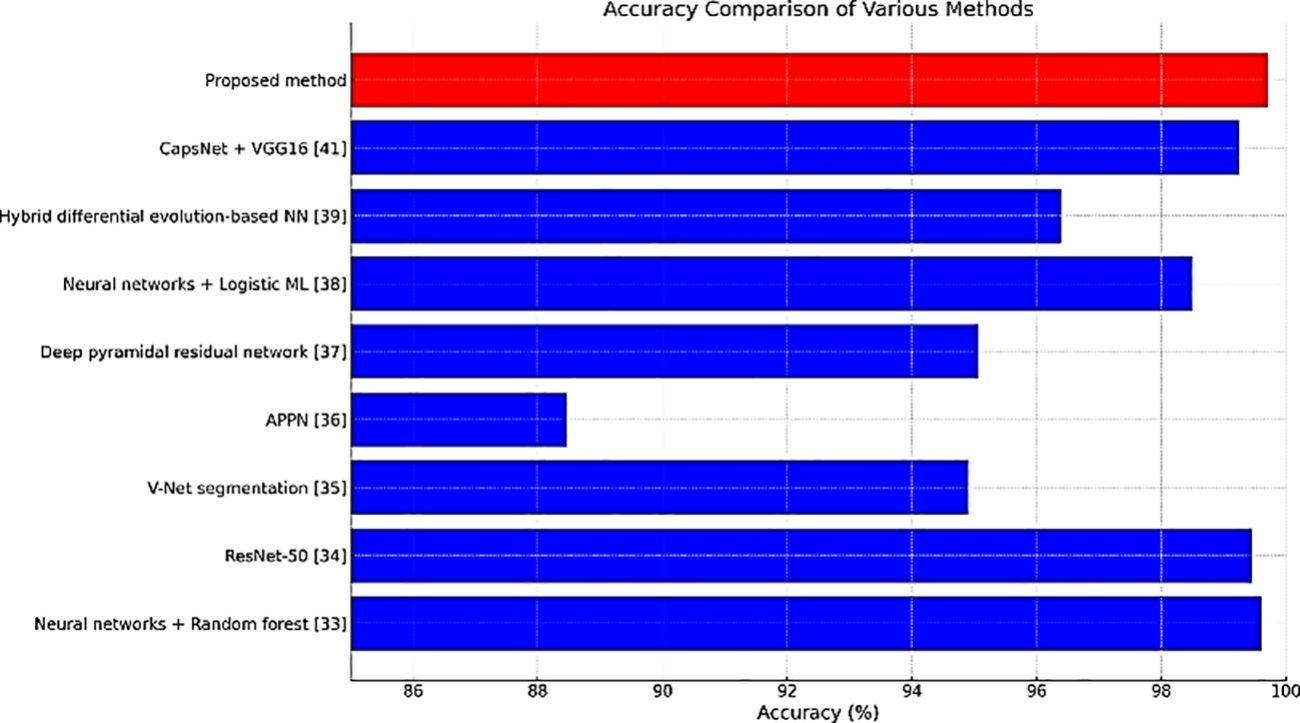
Accuracy comparison plot.

**Figure 12:**
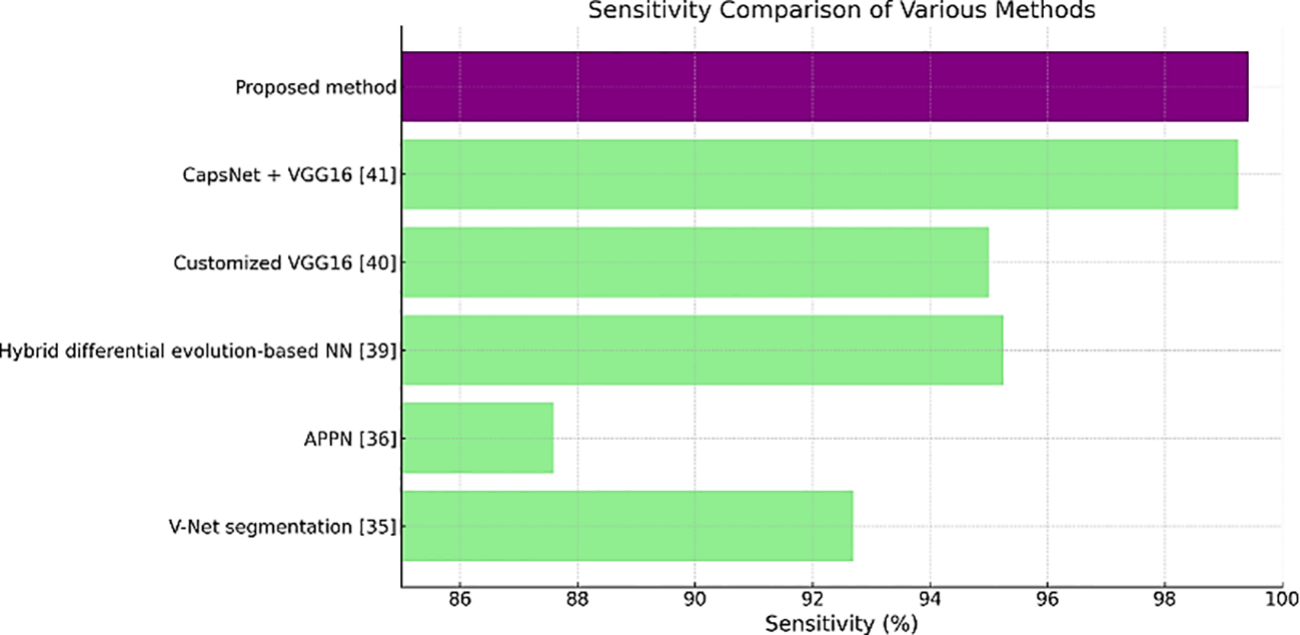
Sensitivity comparison plot.

**Figure 13:**
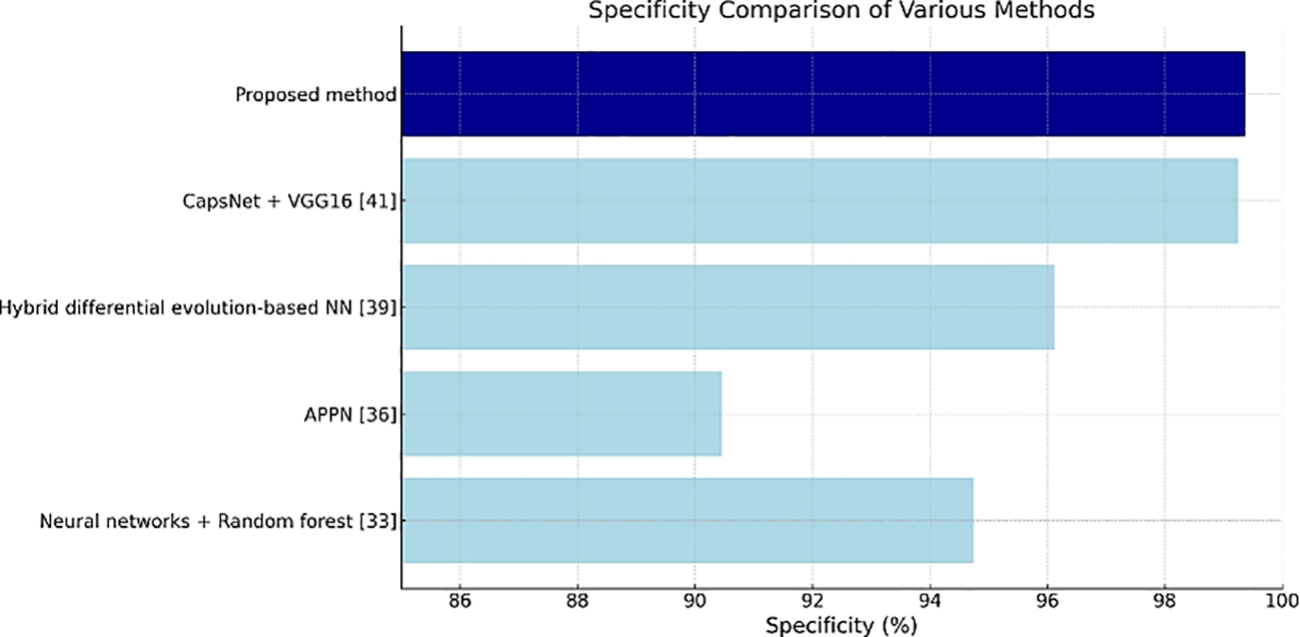
Specificity comparison plot.

**Figures 11**–**13** are bar plots extracted from **Tables 2**–**4**, respectively, providing a visual representation of the numerical data. This makes it easier to compare how different measures of performance across methods are fairing, thus evidencing that the proposed method outperforms others on grounds of accuracy, sensitivity and specificity.

**Figure 14:**
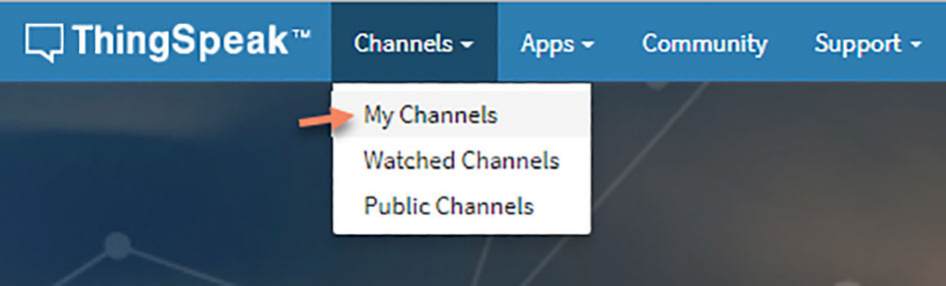
ThingSpeak GUI.

**Figure 15:**
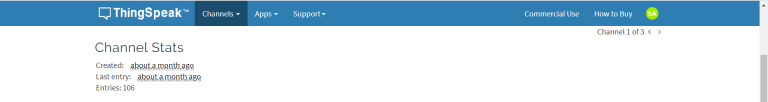
Data Transmission through ThingSpeak.

Finally, the obtained result is transmitted to ThingSpeak and is shared with the authorized personnel only to the corresponding mobile device or Personal Computers. **Figure 14** shows the ThingSpeak GUI, and **Figure 15** shows how the data are shared.

ThingSpeak, with its cloud-based IoT analytics platform (42), is known as the critical part of this research methodology in the detection and classification of lung nodules. In addition to its direct use in this project, ThingSpeak is working toward practical applications in medical imaging and healthcare provided by a broader technological frontier. In the context of this research, ThingSpeak connects applicable parameters and martial statistics to authorized personnel so that they all always remain updated, even if they are far from the site. This functional capacity allows healthcare professionals to perform their responsibilities remotely by monitoring patient data, working interactively concerning diagnosis and treatment decisions, and enhancing workflow efficiency. The methodology is built on the basis of real-time visualization of data and analytics of the ThingSpeak feature. As a result, it broadens the scope of accessibility and helps in better decision making through informed analysis in medical imaging.

In the case of ThingSpeak, the perennial future in healthcare promises to be full of opportunity. With the growth of IoT applications, ThingSpeak will be able to extend its functionalities and use data analysis, predictive modeling, and subsequent decision support systems to help improve the delivery of health services. The possible applications could be facilitating home-based patient monitoring, videoconferencing diagnoses, and health data collation and control for the general public. On top of mind, machine learning and AI algorithms already installed in the ThingSpeak realm can make the latter more efficient and useful in healthcare. Such developments could revolutionize medical diagnosis through the automation of abnormal detection, machine learning for disease forecasting, and the development of individualized treatment plans based on the unique data of patients.

In this effort, IoT integration is crucial since it enables real-time communication of diagnostic results via platforms such as ThingSpeak. This connectivity enables the seamless flow of data between diagnostic systems and healthcare practitioners, allowing for immediate analysis, feedback, and collaborative decision-making regardless of location. The incorporation of IoT in our method not only improves the efficiency of lung cancer diagnoses, but it also opens up new avenues for remote healthcare and telemedicine applications.

The suggested system uses IoT to continually monitor patient data, warn medical professionals in real time about significant findings, and keep a historical database of diagnostic information for continuing patient care. This feature is especially useful in situations where access to specialists is limited, as it enables prompt intervention and better patient outcomes. Furthermore, IoT integration enables scalable systems in which data from many diagnostic devices can be gathered, evaluated, and acted upon inside a single system, hence improving overall lung cancer care management.

This research provides an accurate technique for diagnosing lung nodules. All the advanced methods, such as histogram equalization, the Colliding Bodies Optimization (CBO) algorithm, local binary pattern (LBP) feature extraction, and DenseNet CNN classification, were applied. With this holistic strategy, the resulting data are credible and trustworthy as the quality of images is improved, essential features are extracted, and efficient tumor classification becomes possible. Additionally, ThingSpeak, as an IoT analytic platform interfaced with data streams, makes information transmission and collective decision-making among doctors effortless, facilitating remote access to critical information and timely care for patients.

The presented methodology, in addition to these results, is promising, but some limitations exist that must be considered. The validity of the current results may be limited by the particular dataset used for the experiments, a feature requiring extension of these studies considering different sets of datasets and clinical situations. Furthermore, the computational complexity might be a problem if a deep network algorithm such as DenseNet CNN is applied, which involves intensive resthisce requirements and processing time. Addressing the challenges of sensitivity to input parameters and the need for model interpretability for the integration of deep learning into clinical practice are the parameters that demand an objective and accurate assessment. Overcoming these restrictions by ongoing research and development activities will be a matter of great concern for attaining the best use and spread of this methodology in healthcare situations.

## 6 Conclusion

With the current segmentation approaches, early identification of cancer is quite challenging. Survival rates for lung cancer patients can be improved with early detection and accurate diagnostics. Malignant and benign tissues can be distinguished on CT images using a convolutional neural network (CNN). Traditional or machine learning algorithms cannot perform feature engineering as deep learning algorithms can. This analyses the data to look for related qualities and includes them so that learning can proceed more quickly. spatial coherence in the input is exploited. Images are preprocessed, and later, the feature selection process leading to feature extraction is performed before training and testing. However, diagnosing lung cancer by radiologists is challenging and time-consuming, often requiring significant expertise and careful analysis. The proposed method is denoted as CBO+DenseNet CNN, in which CBO is used for the optimal solution, and the densely connected CNN (DenseNet) deep learning method, in which the tumors are classified as benign or malignant. This study uses a wide range of statistical variables for comparison purposes. The collected data are sent to remote linked devices via the IoT module cloud for evaluation and future analysis. This model could be improved in the future so that it cannot only tell us whether a patient has cancer but also tell us where the tumthiss are located.

## Data Availability

The original contributions presented in the study are included in the article/supplementary material. Further inquiries can be directed to the corresponding author.
